# Optimization of Cladding Diameter for Refractive Index Sensing in Tilted Fiber Bragg Gratings

**DOI:** 10.3390/s22062259

**Published:** 2022-03-15

**Authors:** Sanzhar Korganbayev, Marzhan Sypabekova, Aida Amantayeva, Álvaro González-Vila, Christophe Caucheteur, Paola Saccomandi, Daniele Tosi

**Affiliations:** 1 Politecnico di Milano, 20156 Milan, Italy; sanzhar.korganbayev@polimi.it (S.K.); paola.saccomandi@polimi.it (P.S.); 2 School of Medicine, Nazarbayev University, Nur-Sultan 020000, Kazakhstan; marzhan_sypabekova@baylor.edu; 3 School of Engineering and Digital Sciences, Nazarabayev University, Nur-Sultan 010000, Kazakhstan; aida.amantayeva@alumni.nu.edu.kz; 4 Baylor Research and Innovative Collaborative, Baylor University, Waco, TX 76704, USA; 5 Electromagnetism and Telecommunication Department, University of Mons, 7000 Mons, Belgium; alvaro.gonzalezvila@umons.ac.be (Á.G.-V.); christophe.caucheteur@umons.ac.be (C.C.); 6 Biosensors and Bioinstrumentation Laboratory—National Laboratory Astana, Nur-Sultan 010000, Kazakhstan

**Keywords:** optical fiber sensors, refractive index sensing, tilted fiber Bragg grating, chemical etching, etching optimization

## Abstract

This work presents an experimental investigation of the effect of chemical etching on the refractive index (RI) sensitivity of tilted fiber Bragg gratings (TFBGs). Hydrofluoric acid (HF) was used stepwise in order to reduce the optical fiber diameter from 125 µm to 13 µm. After each etching step, TFBGs were calibrated using two ranges of RI solutions: the first one with high RI variation (from 1.33679 RIU to 1.37078 RIU) and the second with low RI variation (from 1.34722 RIU to 1.34873 RIU). RI sensitivity was analyzed in terms of wavelength shift and intensity change of the grating resonances. The highest amplitude sensitivities obtained are 1008 dB/RIU for the high RI range and 8160 dB/RIU for the low RI range, corresponding to the unetched TFBG. The highest wavelength sensitivities are 38.8 nm/RIU for a fiber diameter of 100 µm for the high RI range, and 156 nm/RIU for a diameter of 40 µm for the small RI range. In addition, the effect of the etching process on the spectral intensity of the cladding modes, their wavelength separation and sensor linearity (R^2^) were studied as well. As a result, an optimization of the etching process is provided, so that the best trade-off between sensitivity, intensity level, and fiber thickness can be obtained.

## 1. Introduction

Tilted Fiber Bragg Grating (TFBG) is a periodic modulation of the refractive index of the core of an optical fiber, photo-inscribed at a certain angle with respect to the fiber radial axis [1]. While a standard fiber Bragg grating (FBG) behaves as an optical narrow-band notch filter [2], the transmission spectrum of a TFBG reveals a collection of backward-propagating cladding mode resonances. In addition to the typical temperature and strain sensitivities, these structures are able to detect variations in the refractive index (RI) of the medium surrounding the optical fiber [3]. TFBGs have seen a substantial development as a sensing technology in the last few years, thanks to their association with different coating materials and their ability to measure and quantify multiple parameters [4,5,6,7,8]. 

Examples of applications in mechanical sensing include optical fiber accelerometers [9], cantilever-based micro-displacement sensors [10], and even three-dimensional shape sensors based on orthogonal TFBGs [4]. However, optical fiber biosensing is the field where TFBGs showcase their highest potential [11]. The biofunctionalization process consists of immobilizing bioreceptors on the outer surface of a TFBG (using, for example, a thin metallic film coating or a silanization process) and performing a specific measurement of a biological analyte. In this configuration, TFBGs encode the RI information entirely in the cladding modes, as these resonances shrink or enlarge as the RI increases or decreases. Several research works have documented TFBG-based biosensors for the detection of cancer biomarkers [12,13], cardiac biomarkers [14], or protein-based analytes [15], among other immunosensing applications [16].

Typical TFBGs used for biosensing operate at infrared wavelengths on standard single-mode fibers (SMF) and with a tilt angle between 5° and 15°. In this way, their optical spectrum can be demodulated using commercial FBG interrogators or optical spectrum analyzers operating in the third optical transmission window. Additionally, a metallic thin film can be deposited on the TFBG surface to exploit plasmonic effects that occur when the P-polarized cladding modes are excited within the grating. On a surface plasmon resonance (SPR) biosensor [17], this film has the dual purpose of hosting bioreceptors and propagates the corresponding plasmon wave [16]. Plasmonic TFBGs provide higher sensitivity and a lower detection limit [18] when compared to bare TFBGs. 

Another method to improve the performance of a TFBG is the use of chemical etching of the fiber cladding, forming an etched TFBG (eTFBG). This structure was previously reported for protein sensing [15] and showed a significant sensitivity improvement over a standard TFBG. The spectral properties of an eTFBG are similar to the properties of the original grating, but the wavelength spacing between cladding modes tends to change as the etching process progresses and the fiber cladding becomes thinner. Among their advantages, eTFBGs are based on standard TFBGs, so the same photo-inscription setup can be used for their fabrication. The optical spectrum of an eTFBG is polarization-independent and, unlike other TFBG configurations, does not require any additional polarizer or polarization controller [15]. Additionally, the cladding modes of an eTFBG span just a few tens of nanometers, and their interrogation can be carried out using standard equipment. eTFBGs encode the RI information in the cladding modes rather than in a core mode as for an etched FBG [19], so it is possible to obtain significant sensitivity ratings without completely depleting the cladding, hence maintaining most of the mechanical stability of the original fiber. Finally, by independently analyzing the Bragg mode, which is RI-insensitive, it is possible to compensate for temperature fluctuations.

In this work, the properties of eTFBGs targeting RI-sensing applications are experimentally investigated. Since the eTFBG behavior depends on the etching process itself, the sensitivity of the grating (both in terms of wavelength shift and intensity change), as well as the spectral intensity levels of the cladding modes, which are studied in every step of the process. The results allow the etching levels for eTFBGs to be optimized, hence providing the best trade-off between sensitivity, intensity level and fiber thickness.

## 2. Materials and Methods

### 2.1. Chemicals

Hydrofluoric acid (HF) was purchased from Sigma Aldrich (Darmstadt, Germany). D-Sucrose was purchased from Thermo Fisher Scientific (Runcorn, UK). Absolute ethanol was purchased from Aidabul Distillery (Kokshetau, Kazakhstan). Etching and calibration processes were conducted in a chemical fume hood at room temperature (±1 °C).

### 2.2. TFBG Photo-Inscription 

The photo-inscription of the TFBGs was carried out using the well-known phase-mask technique [20]. An excimer ArF laser emitting at 193 nm, the desired phase-mask and all the necessary optics were integrated in a Noria FBG Manufacturing System from NorthLab Photonics. A hydrogen-loaded photo-sensitive single-mode optical fiber (PS-1250 from Fibercore UK) was appropriately placed in the system and the photo-inscription parameters were set to a pulse energy of 5 mJ, a repetition rate of 50 Hz and a set of 3 bursts of 7500 pulses per TFBG. A custom phase-mask with a tilted holographic pattern and a pitch of 1078 nm was chosen in order to obtain a TFBG operating at the third optical transmission window. As a result, a set of 1 cm-long TFBGs was produced, exhibiting a tilt angle of 10°. Once the photo-inscription process was finished, the gratings were thermally annealed at 100 °C for 24 h to remove the residual hydrogen content still present in the optical fiber.

### 2.3. TFBG Interrogation

TFBG interrogation was carried out by a previously reported method [15] in which the optical spectrum is measured using an optical backscatter reflectometer (LUNA OBR 4600 from LunaInc, Blacksburg, VA, USA) with a resolution of 8 pm and a wavelength window between 1525 nm and 1610.5 nm. One end of the TFBG was connected to the OBR through a pigtail of SMF. The other end was spliced using a standard optical fiber splicer (Fujikura 12-S, SMF-SMF splicing mode) to another pigtail of SMF, which was connected to a gold reflector (LunaInc, Blacksburg, VA, USA). As a result, the transmission spectrum of the TFBG is reflected to the interrogator so that the overall “transmission-reflection” spectrum can be measured [21]. This setup allows the cladding modes from the TFBG to be analyzed, with the aim of obtaining information about the RI variation of the outer medium.

### 2.4. TFBG Chemical Etching

**Figure 1:**
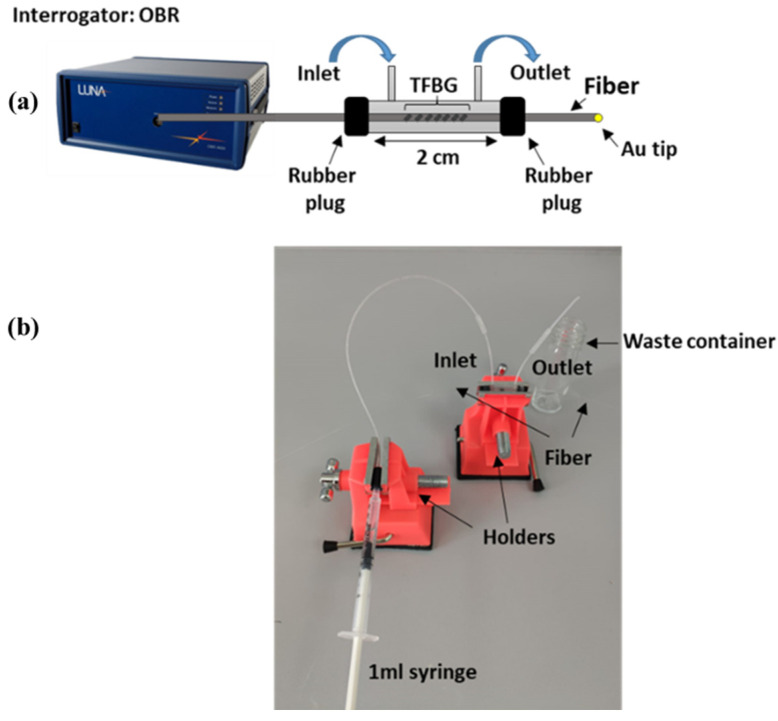
Experimental setup for fiber etching: (**a**) Schematic overview of the setup; and (**b**) Corresponding real image: TFBG was immersed in a closed compartment (cylinder) with 48% HF (during etching stage) or with different sucrose solutions (during RI calibration). The container had two ports: an inlet where the sample was injected using a 1 mL syringe and an outlet which was linked to the waste container.

The etching process was adapted from the previous work [15] by implementing some modifications in the setup. The TFBG was etched by immersing the grating region in a solution of 48% HF in a container located inside a chemical fume hood (Waldner Secuflow airflow controller, ceiling-bench mounted) at room temperature. The etching was conducted in a closed compartment (cylinder) fixed with rubber plugs at each end. The container had two ports: an inlet where the sample was injected using a 1 mL syringe and an outlet, which was linked to the waste container. The container allowed the integrity of the etched TFBG to be maintained during the rinsing steps since the diameter of the fiber becomes thinner after every immersion in HF. Additionally, the container allowed HF vaporization to be avoided during the etching process. The TFBG region of the fiber was placed inside this cylinder, as shown in Figure 1. Before the etching process, the surface of the fiber inside the container was cleaned with ethanol and allowed to air dry. Then, the fiber was incubated in HF solution until the spectrum started to change. After each etching step, the fiber was rinsed thoroughly with dH_2_O several times to remove remaining HF residues, and it was subsequently calibrated using sucrose control solutions with known RI. 

**Figure 2:**
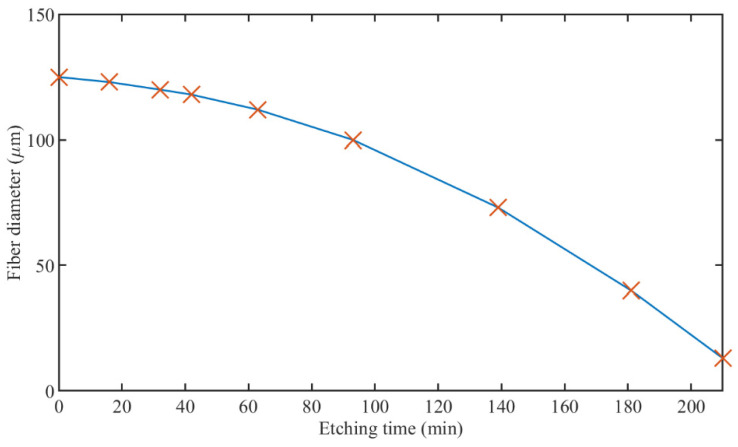
Evolution of TFBG diameter after etching in 48% HF solution. Markers show diameter values after each etching step; blue line—quadratic fitting of the curve.

Calculation of the fiber diameter after each etching step was based on a previously reported diameter estimation approach, where the final diameter was measured using a Leica DM4000 B Digital Microscope with 20×/0.50 objective, and a step-evolution of the diameter was estimated using quadratic fitting [15], as shown in Figure 2.

### 2.5. RI Calibration and Spectral Analysis

Sucrose was used for the preparation of solutions in two RI ranges: the first one with high RI variation (from 1.33679 RIU to 1.37078 RIU; RIU: refractive index units), and the second with low RI variation (from 1.34722 RIU to 1.34873 RIU). The wider range extends for 3.4 × 10^−2^ RIU, which mimics the operation of most biosensors; the second range spans 1.5 × 10^−3^ RIU, which represents a sensor operating on a small-signal analysis, such as biosensors close to the limit of detection [22]. The RI of each solution was measured using an automatic digital refractometer (Anton Paar, Inc., USA, Abbemat 300). TFBG and eTFBG calibration with varying RI were performed by completely injecting the solution inside the container where the TFBG was placed using a 1 mL syringe. Calibration for both RI ranges was carried out after each step of the etching process to investigate the spectral properties and their relation to the etching levels.

Both wavelength and intensity of the cladding modes were tracked using a feature tracking method implemented in MATLAB^®^ (Mathworks Inc., UK) and their variation with regard to the etching process was evaluated. Then, wavelength and intensity sensitivities for both RI ranges were calculated, as well as the coefficient of determination (R^2^ value) of the fitted data. 

## 3. Results

**Figure 3:**
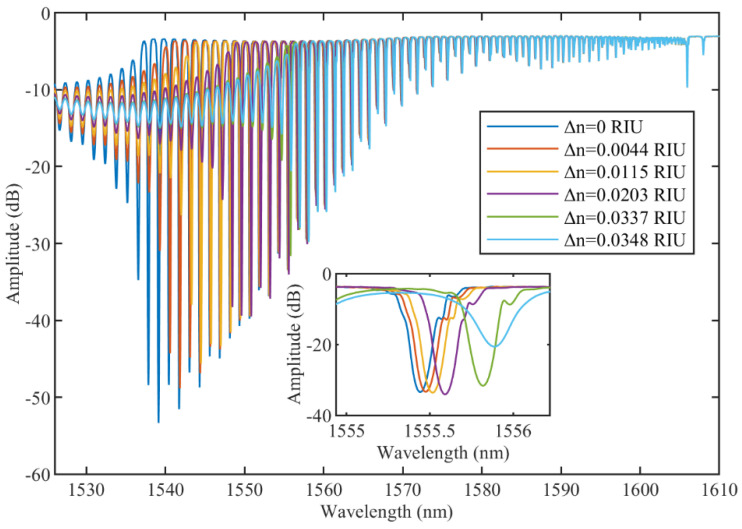
Spectral evolution of unetched TFBG subjected to surrounding RI variations. Inset reports effect on the cladding mode near 1555.5 nm.

The response of the unetched TFBG to RI variations was studied, as can be seen in Figure 3. It should be noted that for the high-order cladding modes (which have lower wavelength values), amplitude changes are more significant than for the low-order modes. Indeed, the increase in RI leads to the gradual disappearance of the modes since they are no longer totally internally reflected by the cladding boundary and reach the cut-off condition [1]. The inset of Figure 3 shows a detailed view of one mode of the TFBG and how it changes due to the variation of the refractive index of the surrounding medium. For minor RI variations, in this case less than 0.0203 RIU, only wavelength shift is observable, while amplitude change corresponds to higher RI variations.

**Figure 4:**
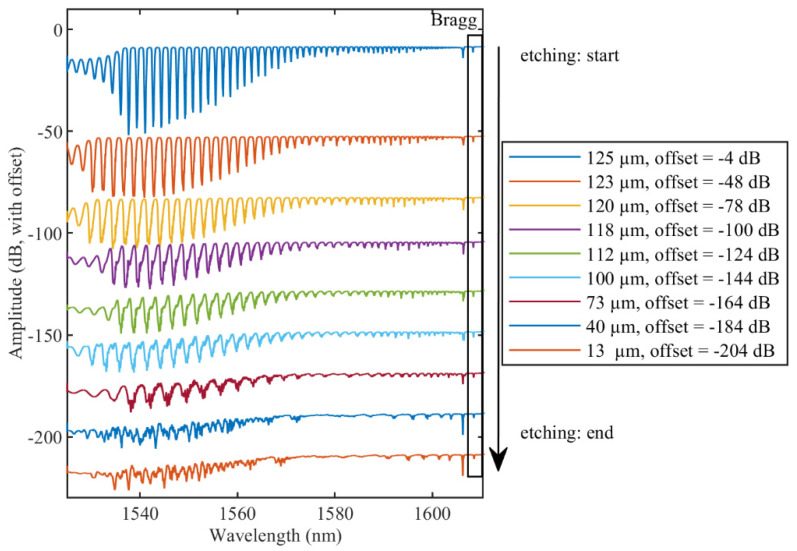
Evolution of TFBG transmission spectrum during the optical fiber etching. Rectangular area shows the Bragg mode of the TFBG. Offset has been introduced for clarity of spectra plotting on one graph.

Then, etching of the TFBG was performed stepwise to evaluate the sensing properties of TFBGs with different optical fiber diameter values. The spectral evolution as a function of the etching process is shown in Figure 4. The fiber diameter decreased due to etching steps from 125 µm to 13 µm. It worth noting that the spectra are provided with an offset for clarity of spectra plotting on one graph. Indeed, without considering an offset, the maximum amplitude value for the unetched TFBG is −5 dB and the minimum is −48 dB, while the etched TFBG (14 μm) has the maximum amplitude of −5 dB and the minimum of −22 dB. A smaller diameter leads to a decrease in the number of modes, due to the mode escaping to the surrounding medium. For a fiber diameter below 40 µm, most of the cladding modes are no longer distinguishable, and only few modes in the range of 1590–1605 nm can be used for interrogation in both RI ranges.

**Figure 5:**
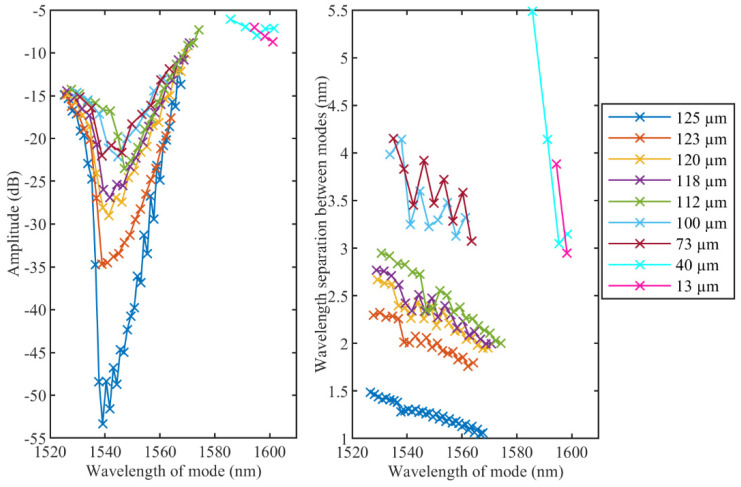
Evolution of amplitude levels (**left**) and wavelength spans (**right**) of cladding modes during etching process. Only modes which are distinguishable for both RI variation ranges are illustrated.

The etching process changes the spectral amplitude of the cladding modes and the wavelength span between adjacent modes [23]. Figure 5 shows the dependence of both parameters on the wavelength of the modes themselves. As can be seen, the minimum amplitude of the unetched TFBG is equal to −53.3 dB, while an eTFBG with a fiber diameter of 73 µm reaches roughly −22 dB. When fiber diameters of 40 µm and 13 µm are reached, just a few modes close to the Bragg wavelength can be measured and their intensity is roughly −8 dB. Moreover, the spectral quality decreases significantly: modes become broader, poorly defined, especially high-order modes (at shorter wavelengths). One of the possible reasons for low spectral quality can be the surface roughness after the etching [23].

Additionally, Figure 5 illustrates the wavelength span evolution corresponding to different fiber diameters. The separation between modes increases from 1 nm to 3 nm for unetched TFBGs and fiber diameters of 73 µm, respectively. The maximum wavelength span experimentally measured is 5.5 nm for the few distinguishable modes, corresponding to a fiber diameter of 40 µm. 

**Figure 6:**
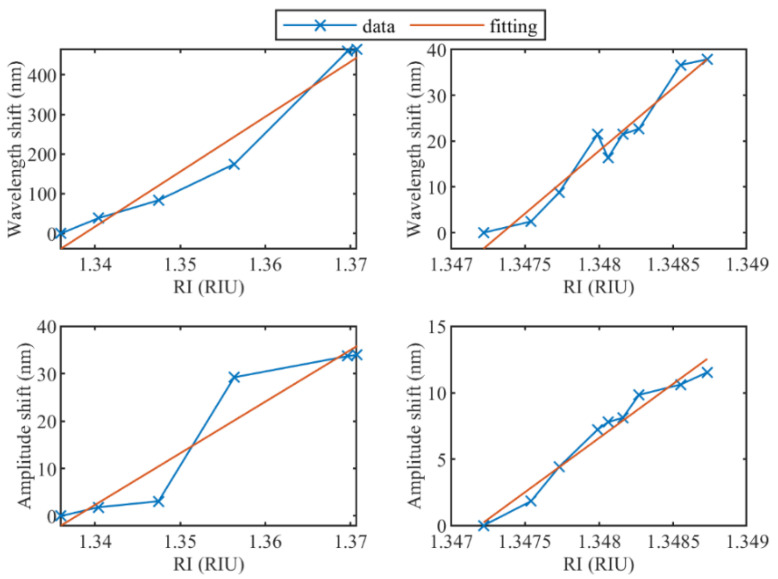
(**left**) Amplitude and wavelength shift for the high RI range from 1.33679 RIU to 1.37078 RIU of unetched TFBG; (**right**) Amplitude and wavelength shift for the small RI range from 1.34722 RIU to 1.34873 RIU of unetched TFBG.

**Figure 7:**
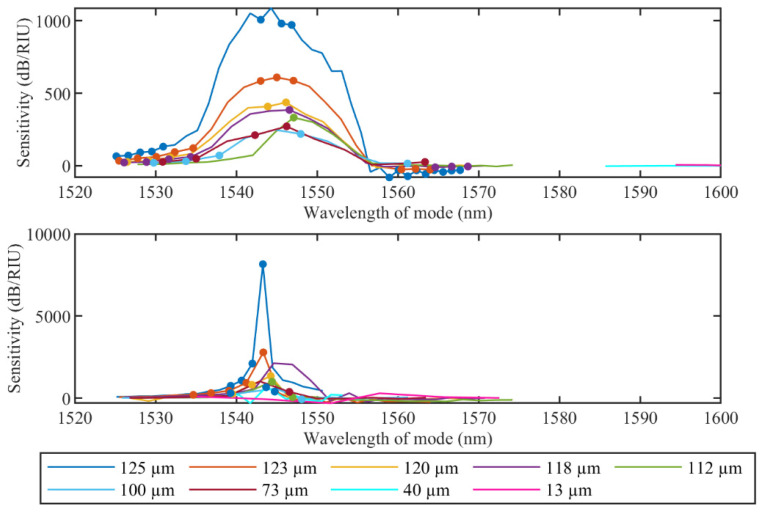
Evolution of the amplitude sensitivity for the high RI range from 1.33679 RIU to 1.37078 RIU (**upper**) and the small RI range from 1.34722 RIU to 1.34873 RIU (**lower**) of cladding modes during etching process. Dot markers show the modes with sensitivity higher than 0.9 (R^2^ > 0.9).

**Figure 8:**
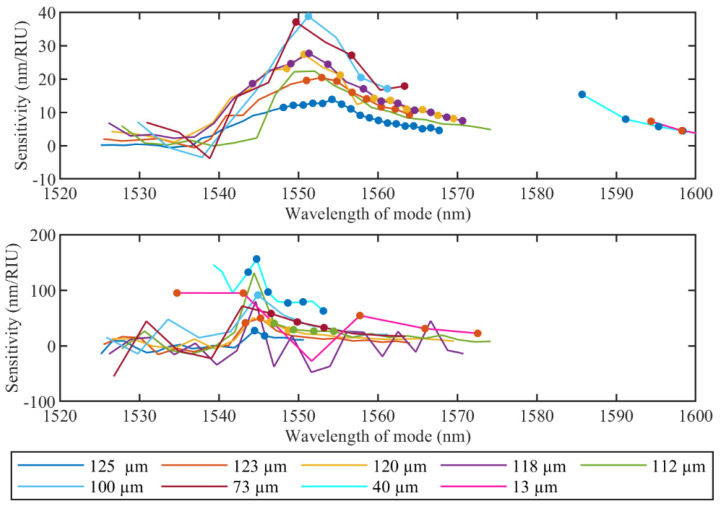
Evolution of the wavelength sensitivity for the high RI range from 1.33679 RIU to 1.37078 RIU (**upper**) and the small RI range from 1.34722 RIU to 1.34873 RIU (**lower**) of cladding modes during etching process. Dot markers show the modes with sensitivity linearity higher than 0.9 (R^2^ > 0.9).

RI calibration has been performed for the high and low RI ranges for all cladding modes. For example, Figure 6 reports shifts of the wavelength and amplitude for both RI ranges and the related fitted linear regression for the most sensitive modes of the unetched TFBG. The same approach has been used for all modes to obtain the sensing information shown in Figure 7 and Figure 8.

Amplitude and wavelength sensitivities of cladding modes after each etching step are illustrated in Figure 7 and Figure 8. In both figures, dot markers show the modes with sensitivity linearity higher than 0.9 (R^2^ > 0.9). As can be seen, maximum amplitude sensitivities for both ranges correspond to unetched TFBGs (1008 dB/RIU and 8160 dB/RIU) and the sensitivity decreases for smaller fiber diameters. It is worth noting that for the high RI variation range, only several modes between 1543 nm and 1547 nm have high sensitivity and good linearity (R^2^ > 0.9), while for the small RI range, only one cladding mode at 1543 nm has high amplitude sensitivity. These results are in correspondence with the theory that the cladding modes with the effective refractive index closer to the surrounding refractive index are the most sensitive for RI variations.

For the wavelength sensitivity, the maximum value for the high RI range is 38.8 nm/RIU for a fiber diameter of 100 µm and 156 nm/RIU with 40 µm for the small range. It is important to highlight that for the small RI range, it was also possible to analyze the “quasi-modes” in the range between 1530 nm and 1560 nm for diameters equal to 40 µm and 13 µm (in Figure 4, they correspond to the two last spectra). As a result, the highest wavelength sensitivity corresponds to 40 µm and is roughly 156 nm/RIU.

## 4. Discussion

The performed experimental investigation shows that the highest amplitude sensitivity corresponds to unetched TFBGs and decreases with etching. In contrast, in the case of the wavelength sensitivity, the highest values are reached for 100 µm and 40 µm fiber diameters. It is worth mentioning that the method used for tracking peaks of TFBG spectra differs from others reported in related research works. For instance, the envelope-tracking method, which evaluates the area between upper and lower envelopes of the TFBG spectrum [24,25] to assess the etching effect on RI sensitivity, is not suitable for small ranges of RI variations because amplitude changes are presented only in several modes and the area does not change significantly. Moreover, this effect is higher after etching due to the decrease in amplitude sensitivity. Considering the fact that small RI detection is gaining more popularity for biosensing applications [12,15,26], an efficient method suitable for small RI range variations has high importance. Another method, which follows individual resonances close to the cut-off region where mode effective index is equal to RI of the surrounding medium, is not effective for etched TFBGs. Indeed, in this case, wavelength separation increases, the dynamic range for each mode becomes bigger, but it becomes more difficult to find a mode near cut-off for a wide range of SRI values. As a result, it is preferable to analyze all modes and compare them for sensitivity values, as was carried out in the presented work.

It is worth discussing the reasons for smaller wavelength and amplitude sensitivities of TFBGs for the higher RI range in this work. With increase in RI of the surrounding medium, more and more high-order modes become no longer totally internally reflected by the cladding boundary (the modes become lossy). As a result, for high RI range analysis, the low-order mode has to be chosen to guarantee its presence for all RI values. However, since such mode is positioned far from the cut-off point, overall RI sensitivity will be low.

Additionally, it is important to mention that a step response of the TFBG sensor is present when the cutoff mode switches from one cladding mode to the adjacent one. This step jump can be considered as an uncertainty in the wavelength measurements. Moreover, high ambient temperature changes can also affect this step jump. However, due to high wavelength separation between the cladding modes (at least 1 nm in Figure 5 right) and low temperature changes in laboratory conditions, this phenomenon was considered insignificant in this work.

Wavelength and intensity tracking play a significant role in optical fiber interrogation systems applied to sensing [27]. Intensity detection [28] is a common method particularly used with plasmonic gratings. However, from a system standpoint, where reliable long-term operation is required, the detection of the wavelength shift is preferable, as it is more robust with respect to the fluctuations of the light sources, fiber twists, and other attenuation effects. In this regard, the eTFBG achieves a significant improvement over the TFBG with the standard fiber diameter, as it allows an almost 4× sensitivity increase obtained with a quite large cladding (~100 µm), which does not compromise the mechanical strength of the fiber. With the help of the figures and outcome of this work, an optimal fiber diameter can be chosen as a function of the desired sensitivity, so the sensor design can be adapted accordingly to the project specifications.

## 5. Conclusions

An experimental investigation was carried out to evaluate the evolution of the TFBG properties after etching, aiming to improve RI sensitivity to the surrounding medium. TFBGs with an initial diameter of 125 µm were etched stepwise in the hydrofluoric acid solution until reaching a fiber diameter of 13 µm. RI calibration was performed after each etching step for different RI solutions in two ranges: the first one with high RI variation (from 1.33679 RIU to 1.37078 RIU), and the second with low RI variation (from 1.34722 RIU to 1.34873 RIU). Wavelength and amplitude sensitivities of all modes, spectral intensity levels of the cladding modes, their wavelength separation and linearity (R^2^) were evaluated for each RI calibration. The highest amplitude sensitivities obtained are 1008 dB/RIU for the high RI range and 8160 dB/RIU for the low RI range, corresponding to the unetched TFBG. However, the highest wavelength sensitivities are 38.8 nm/RIU for a fiber diameter of 100 µm for the high RI range, and 156 nm/RIU for a diameter of 40 µm for the small RI range. The results allow the most suitable etching process to be selected in order to optimize RI sensitivity depending on the interrogation technique required by the application.

## Data Availability

Data underlying the results presented in this paper are not available to the public, but can be obtained from the authors upon reasonable request.
